# Coherent Raman scattering imaging with a near-infrared achromatic metalens

**DOI:** 10.1063/5.0059874

**Published:** 2021-09-14

**Authors:** Peng Lin, Wei Ting Chen, Kerolos M. A. Yousef, Justin Marchioni, Alexander Zhu, Federico Capasso, Ji-Xin Cheng

**Affiliations:** 1 Department of Electrical and Computer Engineering, Boston University, Boston, Massachusetts 02215, USA; 2 John A. Paulson School of Engineering and Applied Sciences, Harvard University, Cambridge, Massachusetts 02138, USA; 3 College of Biotechnology, Misr University for Science and Technology, Giza 12568, Egypt; 4 Department of Physics and Astronomy, University of Waterloo, Waterloo, Ontario N2L 3G1, Canada; 5 Department of Biomedical Engineering, Boston University, Boston, Massachusetts 02215, USA; 6 Photonics Center, Boston University, Boston, Massachusetts 02215, USA

## Abstract

Miniature handheld imaging devices and endoscopes based on coherent Raman scattering are promising for label-free *in vivo* optical diagnosis. Toward the development of these small-scale systems, a challenge arises from the design and fabrication of achromatic and high-end miniature optical components for both pump and Stokes laser wavelengths. Here, we report a metasurface converting a low-cost plano–convex lens into a water-immersion, nearly diffraction-limited and achromatic lens. The metasurface comprising amorphous silicon nanopillars is designed in a way that all incident rays arrive at the focus with the same phase and group delay, leading to corrections of monochromatic and chromatic aberrations of the refractive lens, respectively. Compared to the case without the metasurface, the hybrid metasurface-refractive lens has higher Strehl ratios than the plano–convex lens and a tighter depth of focus. The hybrid metasurface-refractive lens is utilized in spectroscopic stimulated Raman scattering and coherent anti-Stokes Raman scattering imaging for the differentiation of two different polymer microbeads. Subsequently, the hybrid metalens is harnessed for volumetric coherent Raman scattering imaging of bead and tissue samples. Finally, we discuss possible approaches to integrate such hybrid metalens in a miniature scanning system for label-free coherent Raman scattering endoscopes.

## I. INTRODUCTION

Coherent Raman scattering (CRS) microscopy based on coherent anti-Stokes Raman scattering (CARS) or stimulated Raman scattering (SRS) is a label-free, high spatial resolution, and chemical-specific imaging technology.^1–4^ In the past two decades, benchtop CRS microscopes have been demonstrated as powerful laboratory tools for various applications, such as cancer diagnosis,^5,6^ stain-free histopathology,^7,8^ metabolic imaging,^9^ brain imaging,^10,11^ and drug discovery.^12^ Toward translating CRS imaging to the clinic, Hollon *et al.* recently reported a pilot study of brain tumor detection in human tissue slices with a mobile SRS microscope in a surgical suite, showing great potential for stain-free intraoperative histopathology.^13^ However, current benchtop CRS microscopes are bulky and hinder the capacity to image the lesions in human patients and internal organs. To overcome these issues, it is vital to miniaturize the CRS imaging systems, such as developing handheld SRS microscopes^14^ and CARS endoscopes,^15,16^ which directly apply to the human body.

To develop a handheld CRS imaging system, it is essential to design and fabricate high-performance miniature objective lenses that allow high-quality focusing. Because CARS and SRS rely on utilizing two synchronized pulsed laser beams of different wavelengths, namely, pump and Stokes beams, to match a Raman transition, the lenses need to tightly focus the laser beams to the same spot to obtain optimal signals and 3D sectioning resolution. Although commercial achromatic objectives have been widely used in benchtop CRS microscopes, they typically comprise multiple bulky lenses to correct aberrations.^17,18^ For endoscopy, it is challenging to precisely fabricate and align miniature lenses due to their curved configuration and small diameter. As a result, typical endoscope lenses suffer from inferior optical quality and severe monochromatic and chromatic aberrations.^19^

Resulting from their compact footprint and versatile optical properties, metasurface-based optical components, consisting of subwavelength-spaced nanostructures, have found broad applications in miniaturized optical systems,^20–22^ depth sensing,^23–25^ pulse shaping,^26^ and polarization control.^27^ These applications are enabled by the fact that a metasurface is able to simultaneously control transmitted light’s wavefront, dispersion, and polarization. These advantages originate from the constituent nanostructures: each nanostructure has many geometric parameters that are tunable to provide the required phase, polarization, and dispersion properties. Conventional diffractive elements control phase delays by heights, which results in shadow effect lowing transmittance,^28^ while metasurface components have more degrees of freedom^29^ in varying nanostructure shape, leading to high angular efficiency.^30^ For instance, by applying topology optimization and inverse design, the diffraction efficiency of metasurfaces has been increased up to about 95% for high diffraction angles.^31^ By controlling the phase, group delay, and group delay dispersion, achromatic metalenses have been demonstrated in the visible wavelength region.^32–34^ A hybrid metalens that integrates a metasurface with a low-cost singlet refractive lens has shown the ability to eliminate chromaticity as well as other optical aberrations.^35^ Moreover, the development of a diffraction-limited immersion metalens suggested the potential of using a metalens to directly image biological tissues.^36,37^ The aforementioned advances of achromatic metalenses have shown great potential to serve as a high-end miniature objective lens in endoscopic systems.

**FIG. 1.**
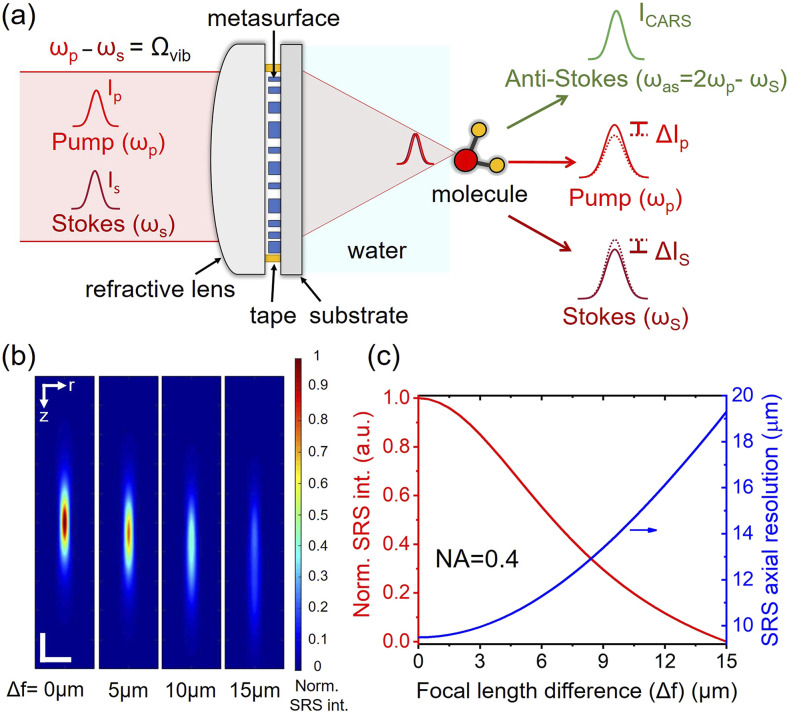
Schematic diagram of a hybrid water-immersion achromatic metalens for CRS imaging and theoretical analysis on the effect of chromatic aberration. (a) Illustration of a hybrid metalens to achromatically focus the pump and Stokes beams. The hybrid metalens consists of a plano–convex glass lens attached to a metasurface comprising *α*-silicon nanopillars. ω_p_ and ω_s_ label the frequencies of the pump and Stokes beams. Ω_vib_ is the targeted Raman transition, which is equal to the energy difference between the pump and Stokes beam (i.e., Ω_vib_ = ω_p_ − ω_s_). Ω_as_ is the frequency of newly generated coherent anti-Stokes light (i.e., Ω_as_ = 2ω_p_ − ω_s_). ΔI_p_ and ΔI_S_ represent the energy loss (i.e., stimulated Raman loss) at the pump beam and the energy gain (i.e., stimulated Raman gain) at Stokes beam before (solid curve) and after (dashed curve) interaction with the molecules, respectively. (b) Simulated SRS intensity when there is a focal length difference (Δf) between the pump beam of *λ*_*p*_ = 800 nm and the Stokes beam of *λ*_*S*_ = 1040 nm. The numerical aperture (NA) of the focusing lens is assumed to be 0.4. (c) Effects of Δf on SRS intensity (red curve) and axial resolution (blue curve). The axial resolution is defined as the full width at half maximum (FWHM) of the longitudinal SRS intensity profile in the focal region.

Here, we report a hybrid water-immersion achromatic metalens that is particularly designed for pump and Stokes wavelengths at the near-infrared region [Fig. 1(a)]. The hybrid metalens consists of a 2-mm-diameter plano–convex refractive lens attached to a 1.5-mm-diameter metasurface. The refractive lens and the metasurface were assembled under a laboratory-built microscope. The hybrid metalens was demonstrated to achromatically focus wavelengths of 800 and 1040 nm with near diffraction-limited performance and used for SRS and CARS imaging at the C–H Raman transition region (i.e., 2800–3100 cm^−1^). By imaging 1-*µ*m polystyrene (PS) beads at 2955 cm^−1^, the hybrid metalens shows a 1.3- and 3.8-times improvement in lateral and axial resolution, respectively, compared with the case of only using the refractive lens. Employing a spectral focusing approach, the hybrid metalens enables spectroscopic forward SRS and backward (epi-) CARS imaging to map and differentiate PMMA and PS beads. Finally, we demonstrate the new capability of the metalens in volumetric CRS imaging of PMMA beads, mouse ear, and ovarian cancer tissue samples. These studies collectively demonstrate a way to develop metalens-based CRS endoscopic imaging systems.

## II. EFFECT OF CHROMATIC ABERRATION ON SRS IMAGING

Using selected wavelengths of the ultrafast pump and Stokes pulses to match a Raman transition, CRS takes advantage of coherent processes to yield a significantly stronger signal than spontaneous Raman scattering spectroscopy.^1^ The pixel dwell time of CRS imaging could be as short as sub-microseconds to enable video-rate chemical imaging.^38^ The pump and Stokes wavelength are defined by $\frac{1}{\lambda_{p}}-\frac{1}{\lambda_{S}}=\Omega_{vib}$, where *λ*_*p*_ and *λ*_*S*_ are the wavelength of the pump and Stokes, respectively, and Ω_*vib*_ is the Raman transition of interest for a given specimen. Figure 1(a) illustrates that when the two laser pulses are focused on Raman-active molecules, three CRS processes occur. CARS light with a new redshifted frequency (*ω*_*as*_) is generated and typically detected by a photomultiplier tube detector with a short-pass optical filter blocking the excitation beams.^39^ In addition, SRS involves the energy transfer from the laser to the molecules, leading to an intensity gain in the Stokes beam and to an intensity loss in the pump. The SRS signal can be extracted via a heterodyne detection approach that detects the subtle energy change in each beam.^40^ The energy loss at the pump beam and gain at Stokes beam are named stimulated Raman loss and stimulated Raman gain, respectively. Both SRS and CARS imaging provide chemical selectivity, whereas CARS has non-resonant backgrounds and spectral distortions that can be removed through multiple approaches, such as phase retrieval algorithms^41,42^ or frequency modulation.^43^

To effectively induce CRS processes, the pump and Stokes pulses need to be focused and overlap well temporally and spatially. Therefore, we designed a hybrid water-immersion achromatic metalens consisting of an *α*-silicon metasurface and an off-the-shelf miniature refractive lens (No. 43-397, Edmund Optics). Before we introduce the design of the hybrid metalens, we first illustrate how chromatic aberration affects the resolution and signal level in SRS imaging. We considered the pump and Stokes wavelengths of 800 and 1040 nm targeting at 2884 cm^−1^, respectively. These near-infrared wavelengths give less photodamage and deeper penetration depth in biological samples.^2^ To calculate SRS signal generation, we assumed that the pump and Stokes are Gaussian beams focused by a lens of NA = 0.4 into a homogeneous dimethyl sulfoxide (DMSO) solution. The SRS signals originate from the overlapped region of the two foci. The SRS intensity along the lateral, ***r***, and longitudinal, ***z***, directions can be modeled as^2^

$$I_{SRS}\left(r,z\right)=C_{0}Im\left(\chi^{\left(3\right)}\right)I_{p}\left(r,z\right)I_{S}\left(r,z\right),$$

where *I*_*p*_(**r**,**z**) and *I*_*s*_(**r**,**z**) are the intensities of the pump and Stokes beams, respectively; C_0_ is a constant; and Im(*χ*^(3)^) is the imaginary part of the third-order nonlinear susceptibility *χ*^(3)^ of the sample. Figure 1(b) shows the cross section of the SRS intensity profiles when the focal length difference between the two beams is 0, 5, 10, and 15 *µ*m. When the two foci are axially separated, the SRS intensity drops significantly. Fitting the SRS intensity profile along the longitudinal direction with a Gaussian function, the blue curve in Fig. 1(c) shows that the SRS axial resolution degrades when the focal length difference increases. Next, we simulated a 1-*µ*m polystyrene (PS) bead placed at the center of the SRS signal and investigated how its SRS intensity is affected by focal length differences. The total SRS signal from the bead is an integral of *I*_*SRS*_(**r**,**z**) over the bead volume. The simulated result given by the red curve in Fig. 1(c) shows that the SRS signal deteriorates dramatically due to chromatic aberration.

## III. PRINCIPLE AND DESIGN OF THE HYBRID WATER-IMMERSION ACHROMATIC METALENS

**FIG. 2.**
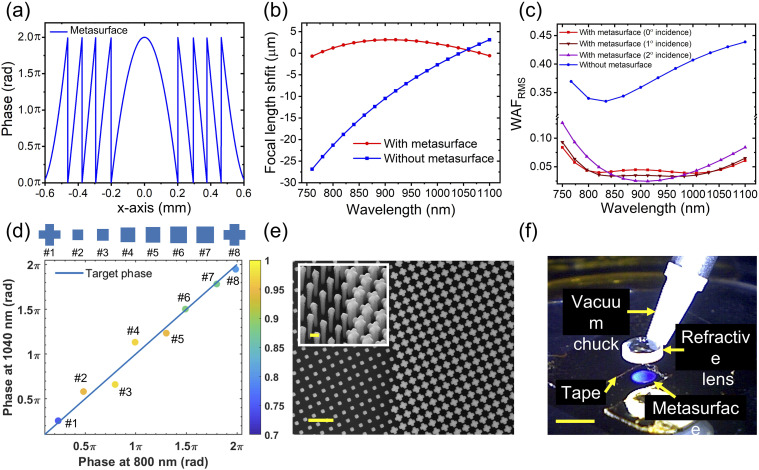
Principle and design of a hybrid water-immersion achromatic metalens. (a) Calculated phase profile of the metasurface along the x-axis passing through the lens center. (b) Simulated focal shift and (c) root-mean-square of wavefront aberration function (WAF_RMS_) with and without the metasurface. The red and brown curves show the WAF_RMS_ at 0°, 1°, and 2° angle of incidence. (d) Designed nanostructures and their phase shifts for *λ*_*p*_ = 800 nm and *λ*_*s*_ = 1040 nm and their transmission at *λ* = 800 nm (shown by colors). The inset illustrates the shape of eight *α*-silicon nanopillars. (e) Scanning electron microscopy (SEM) images from a region of the metasurface. Scale bar: 1 *µ*m. The inset shows an oblique view (scale bar: 200 nm). (f) An image taken while aligning the metasurface to the refractive lens. Scale bar: 2 mm.

The key element of the hybrid water-immersion achromatic metalens (NA = 0.4, 1.5-mm diameter) is the metasurface possessing a phase profile illustrated in Fig. 2(a). Figure S1 shows the parameters of the metasurface and the refractive lens. We used ray-tracing software (ZEMAX OpticStudio, USA) to calculate phases and group delays of all incident rays and adjusted the phase profile of the metasurface and the focal length of the hybrid metalens in such a way that all incident rays arrive at the focus with nearly the same phase and group delay.^35^ These calculations were performed at a wavelength of 904 nm corresponding to the midpoint of the pump and Stokes frequencies. The compensation of group delay leads to a parabolic focal length shift with incident wavelengths [see the red curve of Fig. 2(b)]. Without the metasurface, the plano–convex refractive lens has a focal length monotonically increasing with wavelength [the blue curve of Fig. 2(b)]. In Fig. 2(c), the WAF_RMS_ defined as the optical path difference between the wavefront and an ideal aberration-free wavefront (i.e., a reference spherical surface) with 0°, 1°, and 2° angles of incidence are shown for the cases with and without the metasurface. Not only is the chromatic focal length shift corrected for, but also other aberrations (spherical, coma, and astigmatism) are well-corrected within a field of view of 4°. A WAF smaller than 0.072*λ* is considered as a criterion for diffraction-limited performance.^44^ Considering that the effective focal length of the hybrid water-immersion metalens is 1.86 mm, the field of view covers an area of about 130 × 130 *µ*m^2^ with diffraction-limited resolution.

To implement the metasurface, we built an *α*-silicon nanostructure library consisting of different dimensions of square and cross-shaped nanopillars with 1-*µ*m height. The simulations were carried out by a finite-difference time domain (FDTD) solver (Lumerical, USA) with a periodic boundary (unit cell is 350 × 350 nm^2^). It is worth mentioning that the chosen eight nanopillars are along the 45° line in Fig. 2(d) so that the phase profile shown in Fig. 2(a) can be accurately implemented at both *λ* = 800 and 1040 nm to maintain high diffraction efficiency. The dimensions of the eight nanopillars are listed in Fig. S2. The metasurface was fabricated by e-beam lithography and dry etching following the same recipe reported in Ref. 45. The SEM images from a region of the fabricated metasurface are shown in Fig. 2(e). We assembled the metasurface and the refractive lens under an optical setup (see its schematic in Fig. S3). The refractive lens was held by a vacuum pick-up system and moved to the center of the metasurface [Fig. 2(f)]. During this lateral alignment process, we first marked the center of the metasurface on a camera and then illuminated the refractive lens with a normally incident laser beam such that its focal spot could be seen on the camera. Subsequently, we slightly adjusted the refractive lens’ position until its focal spot on the camera overlapped with the previously marked center of the metasurface. Following this step, we vertically moved the refractive lens to about 20 *µ*m above the metasurface and released the refractive lens. The refractive lens fell on a U-shaped 30-*µ*m-thick double-sided tape (OCA8146-2, Thorlabs). The vacuum pick-up nozzle was used to slightly press the top of the refractive lens to ensure that the refractive lens stuck well to the tape.

## IV. CHARACTERIZATION OF THE HYBRID METALENS

**FIG. 3.**
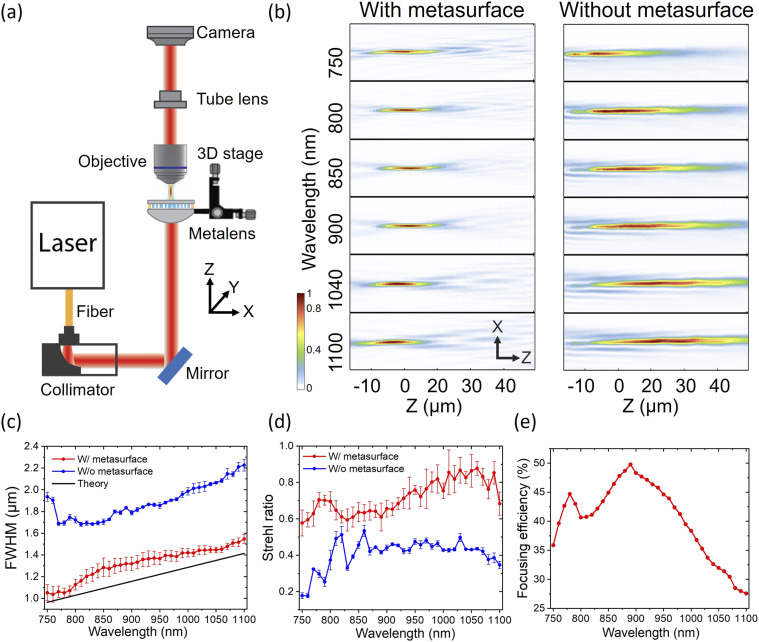
Characterization of the hybrid metalens. (a) Point spread function (PSF) measurement setup, (b) PSFs of the refractive lens with and without the metasurface, (c) FWHM of focus, (d) Strehl ratio of focus, and (e) focusing efficiency of the hybrid metalens, measured from *λ* = 750 to 1100 nm with 10 nm interval. The red and blue curves represent the measurements of the hybrid metalens with and without the metasurface, respectively. For each wavelength in (c) and (d), intensity profiles of the focal spot along horizontal, vertical, and diagonal directions were measured. An Airy disk function was then used to fit the measured intensity profiles to determine FWHMs and Strehl ratios. The average value of these quantities at each wavelength is shown in (c) and (d) as data points, with the standard deviation plotted as error bars. The black line in (c) is the theoretical FWHM of the focal spot calculated when NA = 0.4.

After assembling the hybrid metalens, we built a vertical microscope for characterizing the point spread function (PSF) of the metalens [Fig. 3(a)]. We used a tunable supercontinuum laser (EXTREME from NKT Photonics, LLTF from Photon, etc.) with 5 nm linewidth. The incident beam was collimated by a reflective fiber collimator (RC02F2-P01, Thorlabs) and then focused by the hybrid metalens. The focus was imaged and magnified by a 40× water-immersion objective (LUMPLFLN 40XW, Olympus) and a tube lens (180 mm focal length) onto a camera (DCC1545M, Thorlabs). To obtain a three-dimensional (3D) point spread function (PSF), we sequentially image the focus by moving the hybrid metalens with a translational stage (MT1-Z8, Thorlabs) from z = −40 *µ*m to z = +40 *µ*m at 1 *µ*m intervals and change the incident wavelength from 750 to 1100 nm at 10 nm intervals. Figure 3(b) shows the cross-sectional views of the PSFs at the selected wavelengths for the hybrid metalens and the refractive lens only. The complete PSF images of the hybrid metalens at different wavelengths are shown in Fig. S4. The refractive lens shows spherical aberration and a focal length shift of 30 *µ*m between 800 and 1040 nm, as seen by its long depth of focus and the movement of the peak intensity for each wavelength (Fig. S5), respectively. With the metasurface, the depth of focus becomes shallower and the focal length shift is significantly reduced to 1 *µ*m. We further characterized and compared the FWHMs and Strehl ratios of the hybrid metalens as shown in Figs. 3(c) and 3(d), respectively. The FWHMs of the hybrid metalens are significantly reduced and close to the theoretical values [see the black solid line in Fig. 3(c)]. The Strehl ratios are improved over the whole wavelength range. We noticed that there is an ∼20-*µ*m lateral misalignment (estimated by comparing the measured focal spot profile with simulation, see Fig. S6) between the metasurface and the refractive lens. This translates to an asymmetric focal spot profile leading to larger standard deviation in FWHM and Strehl ratios. Finally, the focusing efficiency was determined by taking the ratio of the focal spot power of the hybrid metalens to that of the refractive lens. The former was measured using the setup shown in Fig. 3(a) by placing a power meter and an iris in front of the camera. The iris had a diameter of about twice the diameter of the central Airy disk to filter out any background light. When measuring the transmitted power of the refractive lens, the iris was removed. The focusing efficiency of the hybrid metalens, as shown in Fig. 3(e), is wavelength-dependent due to the dispersive nature of the nanopillars.

## V. METALENS SRS MICROSCOPE AND ITS PERFORMANCE

**FIG. 4.**
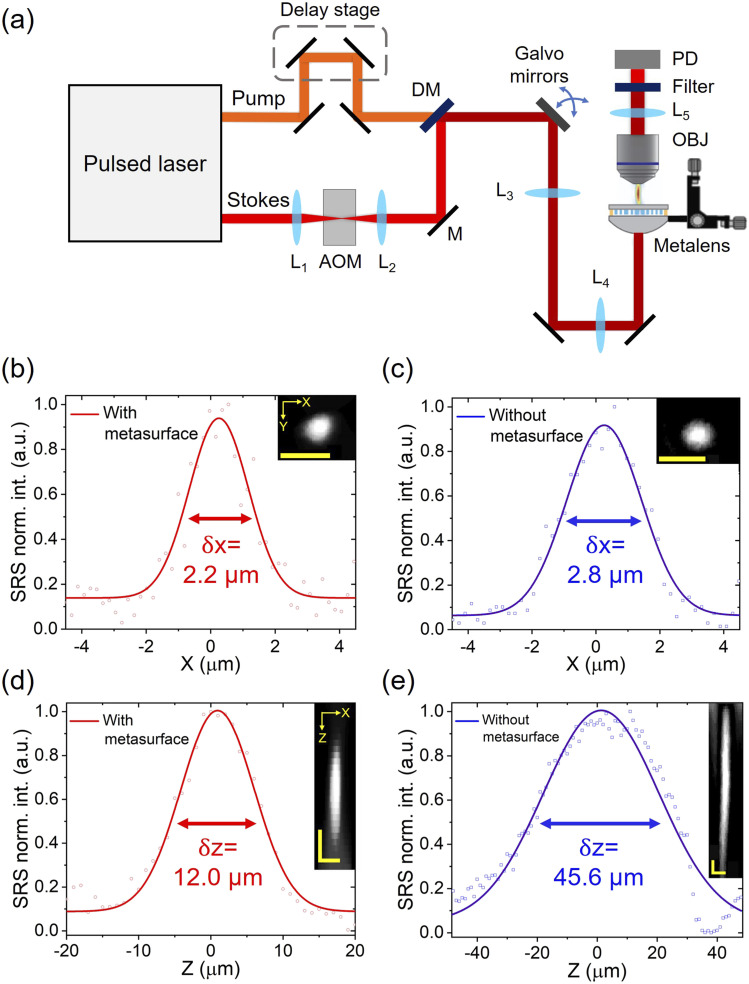
A metalens SRS microscope and its imaging performance. (a) Schematic. AOM: acousto-optic modulator; L: lens; OBJ: objective lens; PD: photodiode; DM: dichroic mirror; M: mirror. (b)–(e) Performance comparison between the hybrid metalens and refractive lens (i.e., without the metasurface) by imaging a 1-*µ*m polystyrene bead at 3060 cm^−1^ Raman shift. (b) and (c) are the intensity profiles along the x-direction, and their inset images are the x–y cross section view of the bead. (d) and (e) are the intensity profiles along the z-direction, and their inset images are the x–z cross section view of the bead. All solid curves are fitted with a Gaussian function. *δ*x and *δ*z are the FWHM of the fitted curves and defined as the lateral and axial resolutions, respectively. All scale bars in the inset images are 5 *µ*m.

We developed a metalens-based SRS microscope and characterized its focusing performance. The experimental setup is illustrated in Fig. 4(a). The laser source is a dual-output ultrafast laser (InSight DeepSee, Spectral-Physics) with a tunable pump beam from 680 to 1100 nm of ∼120 fs duration and a fixed Stokes beam of 1040 nm and ∼220 fs duration, both at 80 MHz repetition rate. The diameters of the output laser beams are about 1.1 mm. To detect the stimulated Raman loss signal, an acousto-optic modulator (AOM, M1250, Isomet) set at 3 MHz modulation frequency is installed at the focal plane of lenses L_1_ and L_2_ (both focal lengths are 100 mm) in the Stokes beam path. The pump beam passes through a motorized delay line stage used for adjusting the temporal overlap of pump and Stokes pulses. A short-pass dichroic mirror (ZT1064rdc-sp, Chroma) is used to combine the two beams. The formation of SRS images is based on raster scanning of the laser focus steered by a pair of galvanometric mirrors (GVS202, Thorlabs). A pair of 200 mm lenses (L_3_ and L_4_) is used to conjugate the galvanometric mirrors and the hybrid metalens held by a 3D motorized stage. A sample is placed at the focal plane of the hybrid metalens. A 40× objective lens (LUMPLFLN 40XW, Olympus, Japan) with 0.8 NA is used to collect the light after the sample. A short-pass filter (No. 64-336, Edmund Optics) is employed to block the Stokes beam. A 30-mm lens is used to focus the pump light to a home-built photodiode (PD).^46^ The PD signal is sent to a lock-in amplifier (MFLI, Zurich Instruments, Switzerland) to demodulate the stimulated Raman loss signals at the pump beam. Before conducting SRS imaging, we used a resolution target to find the focal plane of the hybrid metalens and obtained clear scanning images over an area of 200 × 200 *µ*m^2^ (Fig. S7). Tuning the pump to 798 mm, we used a pure DMSO solution sample to optimize the SRS signal at C–H transition through adjusting the delay between two beams to ensure an optimal temporal match.

We compare the performance of the refractive lens with and without the metasurface by imaging a 1-*µ*m PS bead (No.112, Phosphores Inc.) at a 3060 cm^−1^ Raman shift. The bead sample was sandwiched between two cover glasses and placed at the focal plane of the lens. We imaged the bead by sequentially moving the lens from −45 to +45 *µ*m along the z-axis to form a 3D imaging stack of the bead. The pixel dwell time was 10 *µ*s. The power of the pump and Stokes beams measured before the hybrid metalens was 45 and 130 mW, respectively. Figures 4(b)–4(e) show the x–y and x–z cross section views of the bead and its lateral and axial intensity profiles, respectively. For the refractive lens assembled with the metasurface, the FWHMs along the x- and z-directions [Figs. 4(b) and 4(d)] are 2.2 and 12.0 *µ*m, respectively. In contrary, in the case of the refractive lens alone (i.e., without the metasurface), the FWHMs along the x- and z-directions [Figs. 4(c) and 4(e)] are 2.8 and 45.6 *µ*m, respectively. The results indicate that both lateral and axial resolutions are significantly improved by the metasurface that corrects both chromatic and spherical aberrations.

## VI. METALENS CRS IMAGING OF THE MIXED POLYMER BEADS

**FIG. 5.**
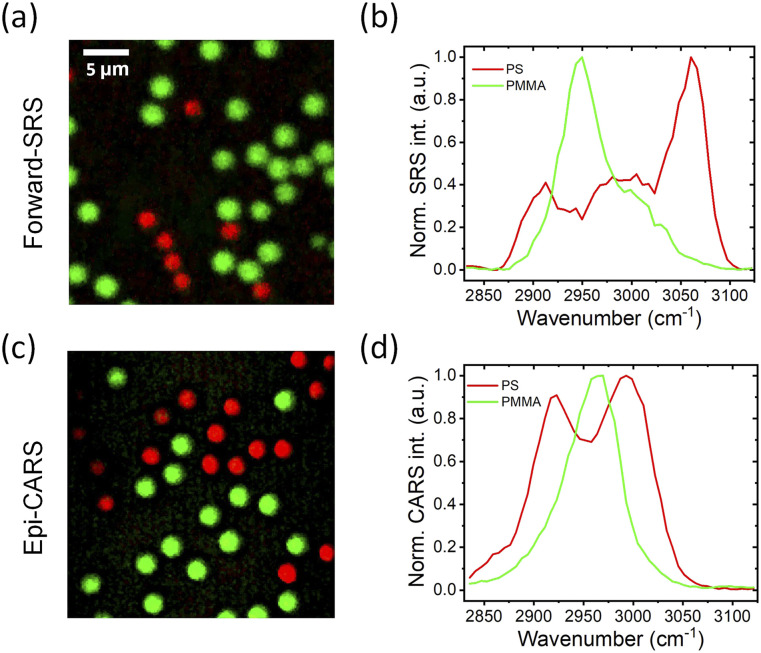
Metalens SRS and CARS imaging of mixed 3-*µ*m polymethyl methacrylate (PMMA) and 2-*µ*m polystyrene (PS) beads. (a) SRS images and (b) spectra of PMMA (green) and PS beads (red) at the forward detection mode. (c) CARS images and (d) spectra of PMMA (green) and PS beads (red) in the backward (epi-)detection mode. Scale bars are 5 *µ*m.

Spectroscopic CRS imaging is able to map and differentiate different chemicals on a sample with high imaging speed. Here, we demonstrate that the hybrid metalens enables spectroscopic SRS and CARS imaging. Employing a spectral focusing approach,^47^ we equally linearly chirped the femtosecond pump and Stokes pulses by placing a 30-cm SF 57 glass rod in the combined path and an additional 15-cm one in the Stokes path (see Fig. S8 for the modified schematic setup). By sweeping the time delay between the two chirped pulses (the pump and Stokes wavelengths were set at 798 and 1040 nm, respectively) with a motorized translational stage, the system can scan the Raman shifts from 2850 to 3100 cm^−1^ to form a spectroscopic imaging stack.^47^ Applying a spectral unmixing algorithm,^48^ Fig. 5(a) illustrates that the two kinds of beads are differentiated based on their characteristic Raman peaks (i.e., PMMA: 2955 cm^−1^ and PS: 3060 cm^−1^) as indicated in Fig. 5(b). Subsequently, we demonstrated the hybrid metalens for backward (epi-)CARS imaging by modifying the setup (see Fig. S8), where a dichroic mirror (Chroma) is installed before the hybrid metalens to reflect the newly generated anti-Stokes light to a photomultiplier tube detector (Hamamatsu). For CRS endoscopes that collect the backscatter photons with a double-clad fiber^15^ or multicore fiber,^16^ CARS imaging may be preferable as the anti-Stokes light with a shorter wavelength (i.e., *λ*_as_ = 647 nm at 2884 cm^−1^) enables more multi-scattering events to bounce back photons from the tissues. Figures 5(c) and 5(d) show the epi-CARS images of PS and PMMA beads and their spectra, respectively. The distortion of the spectra is due to the non-resonant background in CARS imaging and can be corrected using phase retrieval algorithms.^37,38^

## VII. METALENS CRS IMAGING OF LIPID CONTENT IN AN *EX VIVO* MOUSE EAR

**FIG. 6.**
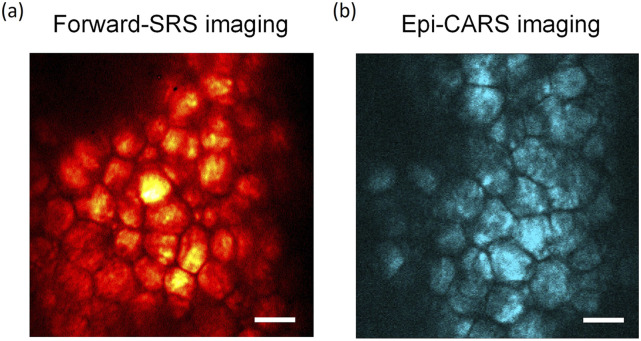
Metalens-based CRS imaging of lipid content in an *ex vivo* mouse ear. (a) Forward SRS image and (b) epi-detected CARS image at 2884 cm^−1^. Scale bar: 50 *µ*m.

Next, we demonstrate that the hybrid metalens enables depth-resolved chemical imaging of mouse ear tissue in both the forward SRS and epi-CARS modes. In the C–H vibrational region (2800–3100 cm^−1^), chemicals such as lipids, proteins, and deoxyribonucleic (DNA) are distinguished based on their Raman spectra.^2,7^ Mapping of lipids and the ratio of lipid-to-protein in lesions by CRS imaging is a rapid and label-free diagnostic approach for atheromatous disease^2^ and brain cancer.^13^ The mouse ear tissue was harvested from a euthanized 8-week-old nude mouse (Boston University Charles River Laboratory) and flattened with a drop of pure water on a coverslip, which was placed on the focal plane of the hybrid metalens. As shown in Fig. 6, the brighter signal in the images represents the lipid-rich region in the cells. The grooves between cells where less lipid concentrated show lower C–H Raman signals. In the supplementary material, videos 1 and 2 show the volumetric SRS and CARS images taken by moving the hybrid metalens from Z = −30 to +30 *µ*m.

## VIII. VOLUMETRIC SRS IMAGING THROUGH THE METALENS

**FIG. 7.**
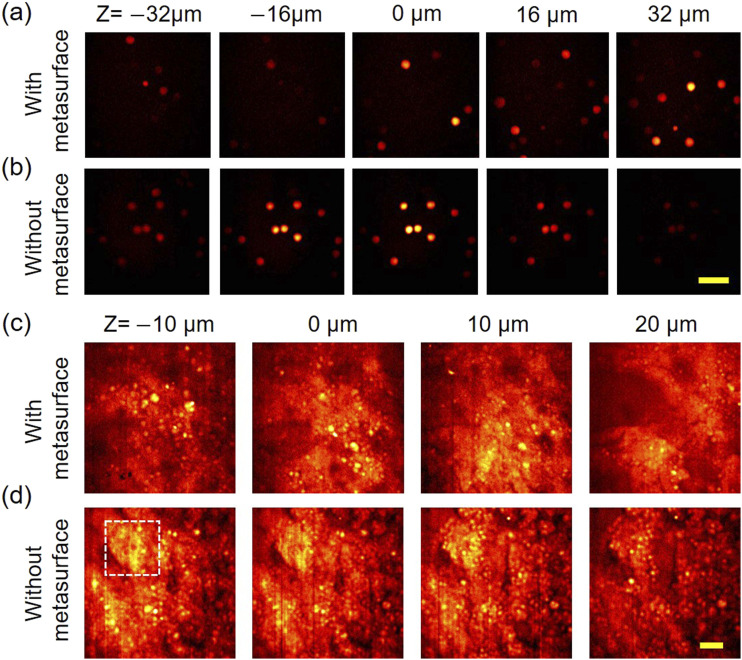
Performance of the hybrid metalens in volumetric SRS imaging PMMA bead and ovarian cancer tissue samples. (a) and (b) are images from the sample of 10 *µ*m PMMA beads imaged (2955 cm^−1^ Raman shift) by the hybrid metalens and refractive lens, respectively. Different depths are labeled. (c) and (d) Ovarian cancer tissue imaged at 2900 cm^−1^ Raman shift by the hybrid metalens and refractive lens, respectively. The bright spots are lipid droplets. Scale bars are 40 *µ*m.

Finally, we investigated the performance of the hybrid metalens in volumetric PMMA bead and ovarian cancer tissue samples. We compared the z-resolution for cases with and without the metasurface. The PMMA bead sample was prepared by 10 *µ*m PMMA beads (Phosphores Inc.) dispersed in 1% agarose gel. A droplet from the gel was sandwiched by two cover glasses with a spacer of double-sided tape (Cat.3136, 3M). The hybrid metalens sequentially imaged the bead sample at different depths at 2955 cm^−1^ Raman shift. The pixel dwell time was 10 *µ*s. Figure 7(a) shows that the hybrid metalens clearly differentiates the beads located at different depths. In comparison, Fig. 7(b) shows that the refractive lens (i.e., without metasurface) barely resolves the beads located at different depths because of its elongated depth of focus. In the supplementary material, videos 3 and 4 show their complete volumetric images from Z = −40 to +40 *µ*m with 2 *µ*m interval. Note that Figs. 7(a) and 7(b) were obtained from the same sample but at different positions because a realignment is required after changing the hybrid metalens and the refractive lens. We then used the hybrid metalens to image the ovarian cancer tissue at 2900 cm^−1^ Raman shift. Such cancer tissue has a complex, three-dimensional lipid structure for showcasing the axial resolution. The tissue sample with 80 *µ*m thickness was sliced from a patient-derived xenograft.^49^ The power of pump and Stokes beams before the hybrid metalens was 45 and 250 mW. The pixel dwell time was 50 *µ*s. In Fig. 7(c), one can see that lipid droplets (bright spots in the images) at different depths can be resolved while moving the hybrid metalens by 10 *µ*m steps. In contrary, in Fig. 7(d), images obtained from different depths are similar and the boundary of lipid droplet is vague (see the area outlined by the white dashed line). The ability to resolve accumulated lipid droplets in cancer tissue holds potential for cancer diagnosis.^5,6^

## IX. DISCUSSION

We have designed and fabricated a hybrid achromatic water-immersion metalens for CRS imaging. The hybrid metalens comprises a low-cost 2-mm-diameter plano–convex lens and a 1.5-mm-diameter metasurface consisting of 1-*µ*m-height *α*-silicon nanopillars. The metasurface was designed and characterized to have nearly diffraction-limited focal spots from *λ* = 800 to 1040 nm, matching typical Raman transitions with a Strehl ratio of >0.7. After verifying the hybrid metalens’ improved focal spot size and depth of focus by the point spread function measurement, we demonstrated the capability of the hybrid metalens in spectroscopic and volumetric CRS imaging of bead and tissue samples, showcasing its promising potential for miniature endoscopic CRS imaging.

Further desired improvements include increasing the focusing efficiency of the hybrid lens. The fabricated hybrid metalens in this manuscript has about 50% peak measured focusing efficiency, which is lower than about the 92% from simulations (Fig. S9). The rest of the transmitted light either goes toward the secondary foci or background light. We attribute the difference between experiment and simulation to the tapered sidewall of the *α*-silicon nanopillar due to deep dry etching, as seen in the inset of Fig. 2(e). Our previous study suggests that a 4° tapering introduces a significant phase error of ∼150° when a nanopillar’s diameter is 220 nm.^50^ This tapering effect may be reduced by fine tuning the ratio of etching gases to the substrate temperature.

An emerging design of miniature CRS imaging probe is based on fiber scanning.^15^ In these setups, the facet of a fiber is held and steered by a piezoelectric tube in a circular or Lissajous scanning pattern. Since the light from the fiber tip is divergent, an endoscopic imaging system usually requires a collimating lens and a miniature objective to form a finite-conjugated system that relays the laser focus from the fiber tip to the sample. The current hybrid metalens was designed as an infinite-conjugate lens, i.e., focusing a collimated beam to a spot. It is possible to design a finite conjugate metalens that focuses light from a fiber to a sample with a single element.^51^ This can further reduce the footprint of a hybrid metalens compared to the systems using only refractive elements.

## SUPPLEMENTARY MATERIAL

See the supplementary material for details of the design parameters of the hybrid metalens and nanostructures, optical setup for assembling the metasurface and refractive lens, measured focal length shift, measured and simulated focal spot profiles to estimate lateral alignment error, scanning transmitted images with the hybrid metalens, detailed schematic setup for the spectroscopic SRS and epi-CARS microscope, simulation of a metasurface optimized for *λ* = 800 and 1040 nm, volumetric SRS and CARS images of mouse ear tissues in supplementary videos 1 and 2 , and volumetric imaging of PMMA beads and ovarian cancer tissues in supplementary video 3 and 4.

## Data Availability

The data that support the findings of this study are available from the corresponding authors upon reasonable request.
